# Porcine Circovirus 2 Deploys PERK Pathway and GRP78 for Its Enhanced Replication in PK-15 Cells

**DOI:** 10.3390/v8020056

**Published:** 2016-02-20

**Authors:** Yingshan Zhou, Baozhu Qi, Yuanxing Gu, Fei Xu, Huahua Du, Xiaoliang Li, Weihuan Fang

**Affiliations:** Zhejiang Provincial Key Laboratory of Preventive Veterinary Medicine, Institute of Preventive Veterinary Medicine, Zhejiang University, Hangzhou 310058, China; yszhou@zju.edu.cn (Y.Z.); qibaozhu@yeah.net (B.Q.); guzi.123@163.com (Y.G.); flying0926@126.com (F.X.); huahuadu@zju.edu.cn (H.D.); xlli@zju.edu.cn (X.L.)

**Keywords:** porcine circovirus 2, ER stress, apoptosis, pathogenesis

## Abstract

Porcine circovirus type 2 (PCV2) infection induces autophagy and apoptosis. These cellular responses could be connected with endoplasmic reticulum (ER) stress. It remains unknown if PCV2 induces ER stress and if autophagy or apoptosis is primary to PCV2 infection or secondary responses following ER stress. Here, we demonstrate that PCV2 triggered unfolded protein response (UPR) in PK-15 cells by activating the PERK/eIF2α pathway without concomitant activation of IRE1 or ATF6. Since ATF4 and CHOP were induced later than PERK/eIF2α, it is clear that persistent PCV2 infection could lead to selective activation of PERK via the PERK-eIF2α-ATF4-CHOP axis. Therefore, PERK activation could be part of the pro-apoptotic signaling via induced expression of CHOP by PCV2. Since PERK inhibition by GSK2606414 or RNA silencing or suppression of eIF2α dephosphorylation by salubrinal limited viral replication, we suppose that PCV2 deploys UPR to enhance its replication. Over-expression of GRP78 or treatment with tauroursodeoxycholic acid could enhance viral capsid expression and/or viral titers, indicating that these chaperones, endogenous or exogenous, could help correct folding of viral proteins. Our findings provide the first evidence that ER stress plays a role in the pathogenesis of PCV2 infection probably as part of autophagic and apoptotic responses.

## 1. Introduction

Porcine circovirus type 2 (PCV2) is the primary causative agent of porcine circovirus-associated disease (PCVAD) [1]. The single-stranded circular DNA genome of PCV2 contains three major open reading frames (ORFs) coding for the replication protein (ORF1) [2,3], capsid protein (ORF2) [4], and a protein with suggested apoptotic activity (ORF3) [5,6] during PCV2 infection. The virus primarily targets the lymphoid tissues, leading to lymphoid depletion and subsequent immunosuppression in pigs [7]. Although much progress has been made toward understanding the pathogenetic aspects of PCV2 infection, such as mechanisms of viral replication [8,9], epidemiology [10], clinical manifestations [11], host immune responses [12] and control strategies [13], there are still many important questions unanswered hitherto with regard to the pathogenesis of PCV2 infection.

Apoptotic cell death could be one of the mechanisms of lymphoid depletion during PCV2 infection [14]. It remains unknown if apoptosis is the direct response of host cells to viral proteins such as ORF3 or capsid protein (Cap) [6,15] or indirect (or even secondary) host cell responses following autophagy or unfolded protein response (UPR). UPR, autophagy and apoptosis have been found to occur in succession in host cells undergoing persistent stresses via a number of common signaling mechanisms shared by these different cellular responses in deciding the cell fate [16].

Eukaryotic cells respond to accumulation of unfolded proteins in the lumen of endoplasmic reticulum (ER) either by UPR or by apoptosis when cytoprotective functions of the ER are overwhelmed [17]. ER stress activates the stress sensors ATF6 (activating transcription factor 6) [18], IRE1 (inositol requiring enzyme 1) [19], and PERK (PKR-like ER kinase) [20], representing three branches of the UPR. All three proximal UPR transducers associate with GRP78/BiP (a chaperone of the heat shock protein HSP70 family) [21] in their inactivate state. Upon perturbation of ER homeostasis, GRP78 dissociates from the UPR transducers to permit their signaling. Each transducer produces a transcription factor (ATF6(N), XBP1, and ATF4, respectively) that activates genes to increase the protein-folding capacity in the ER [22].

Some viruses, such as hepatitis C virus, hepatitis B virus and Varicella-zoster virus, could manage to establish persistent infections by interacting with UPR [23,24,25]. Mounting evidence suggests that virus infection is related not only to UPR but also to autophagy and apoptosis [26,27]. We have found that PCV2 induces autophagy in PK-15 cells by repressing mTOR in a cascade of phosphorylated proteins involving TSC2, ERK1/2, and AMPK [28,29] and that autophagic response seems to enhance viral replication [29]. Several reports indicate that ORF3 and Cap of PCV2 are involved in apoptotic cell death [6,15]. Apparently, a number of important issues remain to be addressed: Do the autophagic response and apoptosis occur independently during PCV2 infection? Is UPR activated in PCV2-infected cells? Is UPR, once activated, linked with autophagy and/or apoptosis? The present study clearly shows that PCV2 induces UPR by activating the PERK pathway and employs PERK pathway and GRP78 to enhance its replication. There is a possible link of UPR with autophagy and apoptosis during PCV2 infection. Further research is warranted to explore the signaling mechanisms governing the switch or transition from UPR to autophagy and/or apoptosis.

## 2. Materials and Methods

### 2.1. Virus and Cell Lines

Porcine circovirus type 2 isolate SY4 strain (PCV2b, GenBank accession No. GU325754) was originally isolated from the lung of a pig with naturally occurring PCVAD in Zhejiang, China. Cell lines PK-15 free of PCV1 contamination were cultured at 37 °C and 5% CO_2_ in complete medium minimal essential medium (MEM, HyClone, South Logan, UT, USA) supplemented with 10% heat inactivated newborn calf serum (Gibco, Grand Island, NY, USA), 1% l-glutamine, 1% non-essential amino acids, 100 U/mL penicillin G, and 100 g/mL streptomycin. Porcine alveolar macrophage cells (PAM, cell line 3D4/31) (ATCC, Manassas, VA, USA) were grown in RPMI 1640 (Gibco, Grand Island, NY, USA) supplemented with 10% heat inactivated newborn calf serum, 1% nonessential amino acids (100×, GIBCO-BRL), and antibiotics (100 U/mL penicillin G and 100 g/mL streptomycin).

### 2.2. Plasmid Construction and siRNA

Porcine grp78 cDNA was prepared by reverse transcription of total PK-15 RNA, followed by a two-step PCR amplification. The gene was subcloned into the BamHI/XhoI sites of pcDNA3.1 (Invitrogen, Eugene, OR, USA) to generate pcDNA3-GRP78. For the epitope tagging, the FLAG sequence was subcloned into the pcDNA3-GRP78 at the ER signal sequence of GRP78 at residue 19. The recombinant plasmid was confirmed by sequencing. siRNAs against GRP78, PERK and control scrambled siRNA were purchased from Genepharma (Shanghai, China). The sense strand of siRNA for GRP78 was 5'-GGGAAAGAAGGUUACUCAUGCAGUU-3'. For PERK silencing, four siRNAs were used as a pool [30]: 5'-CCAGAGAAGTGGCAAGAAA-3', 5'-CGAGAGCCGGAUUUAUUGA-3', 5'-GGAUGAAAUUUGGCUGAAA-3' and 5'-CAGACACACAGGACAAGUA-3'.

### 2.3. Virus Infection, Treatments with Chemicals and Transfection

PK-15 cells and PAM cells were infected with PCV2 at varying multiplicity of infection (MOI) according to the requirements of different experiments. Infection was allowed to proceed for 1 h and the supernatant was then removed. The cell monolayers were rinsed with sterile phosphate buffered saline at pH 7.2 (PBS) to remove unattached viruses and then incubated in the presence of fresh medium at 37 °C for indicated time points when the cells were harvested for SDS-PAGE/Western blotting of target protein molecules related to UPR or for virus titration as described below.

To examine the interaction between PCV2 and host cells or among host cell molecules related to UPR during virus infection, PK-15 cells were transfected with pcDNA3-GRP78 or siGRP78, or treated with the following chemicals: 0.5 mg/mL tauroursodeoxycholic acid (TUDCA, Calbiochem, Darmstadt, Germany) or 10 mM 4-phenylbutyric acid (4-PBA, Santa Cruz Biotechnology, Santa Cruz, CA, USA) (both as ER stress attenuator), 1 μM PERK inhibitor GSK2606414 (Selleck Chemicals LLC, Houston, TX, USA), 50 μM Salubrinal (Selleck) as eIF2α dephosphorylation inhibitor, 2 μM thapsigargin (Abcam, Cambridge, UK) or 10 μM GRP78 inducer BIX (Tocris Bioscience, Bristol, UK). The cells were then infected as described above and cultured in fresh medium in the absence or presence of the same chemical (at the same concentrations for pretreatments), or the corresponding level of the solvent dimethylsulfoxide (DMSO) as control.

pcDNA-GRP78 or siGRP78 or siPERK was delivered into PK-15 cells by transfection with lipofectamine 2000 (Invitrogen). Cells were infected with PCV2 at 24 h post-transfection. All PCV2-infected cells were subjected to further incubation for 36 h or other time points as specified in related figures before being harvested for Western blotting of target molecules related to UPR or for virus titration as described below.

### 2.4. Western Blotting

Western blotting was performed as described previously [29]. The membranes were blocked for 1 h in Tris-buffered saline containing 0.05% Tween 20 and 5% nonfat milk and then probed for 1 h with the following primary antibodies: mouse monoclonal anti-Cap IgG (produced in our laboratory), rabbit polyclonal anti-LC3B (Sigma, St. Louis, MO, USA), rabbit monoclonal antibodies to GRP78, eIF2α phospho and total eIF2α (Abcam, Cambridge, MA, USA), rabbit polyclonal antibodies to IRE1 phospho, ATF6, PERK phospho, PERK, C/EBP homologous protein (CHOP, Abcam, Cambridge, MA, USA), goat anti-ATF4 polyclonal antibody (Abcam), and mouse monoclonal antibodies to β-actin and Flag (MultiSciences, Hangzhou, China). Blots were washed and then incubated for another hour with goat anti-rabbit, anti-mouse or donkey anti-goat horseradish peroxidase-labeled antibodies (KPL, Gaithersburg, MD, USA). The blots were revealed using the ECL Plus detection system under conditions recommended by the manufacturer (Thermo, Marina, CA, USA). Images were captured directly by the Gel 3100 Chemiluminescent and Fluorescent Imaging System (Sagecreation, Beijing, China). Quantification of band density was done using Quantity One software (Bio-Rad, Hercules, CA, USA) with normalization to the β-actin signal. Abundance of interested proteins in various treatments was expressed, where appropriate, relative to that under mock conditions.

### 2.5. Xbp1 Splicing

Activation of IRE1 was determined by measuring splicing of its substrate, the mRNA encoding the XBP1 transcription factor. PK-15 cells were infected with PCV2 for 12 to 36 h. Cells treated with 2 μg/mL tunicamycin (Sangon, Shanghai, China) for 3 h were used as positive control. RNA was harvested using TRIzol reagent (Invitrogen). Total RNA was treated with DNase I (Thermo, Marina, CA, USA) before synthesis of cDNA by reverse transcriptase (The GoScript™ Reverse Transcription System, Promega, Madison, WI, USA). To amplify xbp1 mRNA, PCR was performed for 30 cycles (94 °C for 30 s, 58 °C for 30 s, and 72 °C for 1 min (10 min in the final cycle)), using the primers 5'-GGCAGAGACCAAGGGGAATG-3' and 5'-GGGTCGACTTCTGGGAGCTG-3' with Platinum Taq DNA polymerase (Invitrogen). Fragments of 235 bp and 263 bp, representing spliced (sXBP1) and unspliced XBP1 (uXBP1), respectively, were documented after staining the 2% agarose gel with ethidium bromide.

### 2.6. TUNEL Assay

PK-15 cells were infected with PCV2 in the absence or presence of TUDCA or 4-PBA for 36 h. Cells were fixed with 4% paraformaldehyde solution in PBS for 1 h at room temperature and then permeabilized with 0.1% Triton X-100 in 0.1% sodium citrate solution for 2 min on ice. The TUNEL assay kit (Roche Molecular Biochemicals, Mannheim, Germany) was used to visualize apoptotic cells following the manufacturer’s instruction.

### 2.7. Immunofluorescence

PK-15 cells were infected with PCV2 in the absence or presence of TUDCA or 4-PBA for 36 h. Cells were fixed with pre-cooled 80% acetone at −20 °C for 15 min. After washing with PBS containing 0.05% Tween-20 (PBS-T), the cells were incubated at 37 °C for 1 h with 1:500-diluted monoclonal antibodies to PCV2 capsid protein. The plate was washed three times with PBS-T and incubated with the secondary fluorescein-labeled goat anti-mouse IgG (KPL) at 37 °C for 45 min. The cells were washed and examined for viral antigen under a fluorescence microscope (X81, Olympus, Japan).

### 2.8. Quantification of Virus Titer

Quantification of virus titer was performed as previously described [29].

### 2.9. Cell Viability Measurement

Cell viability was determined using the CCK-8 assay kit (Beyotime, Hangzhou, China) according to the manufacturer’s instructions.

### 2.10. Statistical Analysis

All results in figures were presented, where appropriate, as means ± the standard deviations from three independent experiments and analyzed by using the Student *t* tests.

## 3. Results

### 3.1. PCV2 Infection Induced Unfolded Protein Response via PERK Pathway

**Figure 1:**
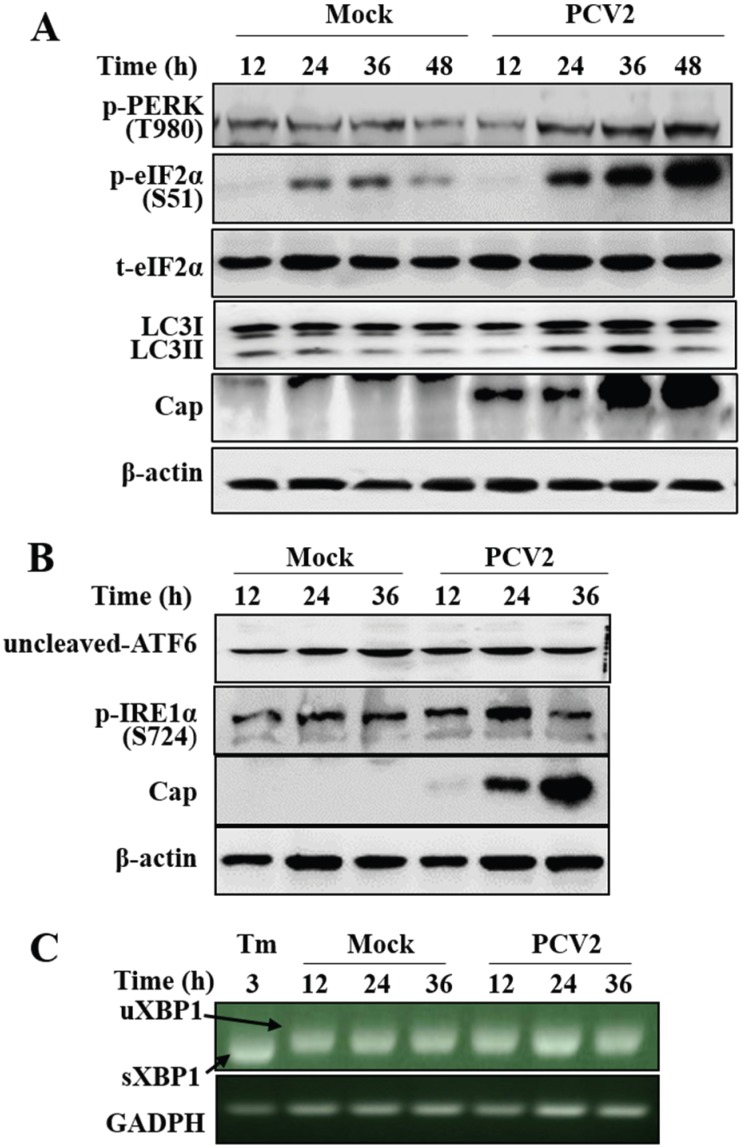
Porcine circovirus 2 infection induced unfolded protein response and autophagy in PK-15 cells. PK-15 cells were infected with PCV2 (MOI = 1) or mock-infected for indicated time points (A, B and C). (**A**) Western blotting of phosphorylated forms of PERK (p-PERK) and eIF2α (p-eIF2α), total eIF2α, autophagy marker LC3-II and PCV2 capsid expression; (**B**) Western blotting of ATF6 and phosphorylated IRE1α (p-IRE1α); (**C**) PK-15 cells were either treated with tunicamycin (Tm) for 3 h or infected with PCV2 (MOI = 1) for indicated time points. Total RNA was extracted and analyzed for splicing of XBP1 (sXBP1 for the spliced, or uXBP1 for the unspliced) visualized on 2% agarose gel after reverse transcription PCR using with XBP1-specific and GAPDH-specific primers. Tunicamycin was used as a positive control for induction of XBP1 splicing (sXBP1) and GAPDH levels as loading control.

We have previously shown that PCV2 infection can employ autophagy to enhance its replication in PK-15 cells [28]. We were wondering whether UPR is involved during PCV2 infection as part of the host cell responses other than autophagy. By examining the three branches of UPR marker molecules PERK/eIF2α, ATF6, IRE1α/XBP1 splicing, we find that UPR did not occur until 24 h post-infection (hpi) as shown by elevation of phosphorylated form of PERK and eIF2α (p-PERK and p-eIF2α) (Figure 1A), a similar time point when autophagy was induced. Since IRE1α might be activated at early phase [31], we collected samples from 12 to 36 hpi for analysis. Figure 1B shows that the phosphorylated form of IRE1 (p-IRE1) was not induced during PCV2 infection, neither was the other UPR sensor ATF6 activated, since cleaved ATF6 could not be detected. Analysis of XBP1 splicing in PCV2-infected cells revealed that no splicing occurred (Figure 1C), while the control cells treated with tunicamycin did exhibit a band of sXBP1 reflecting activation of UPR.

**Figure 2:**
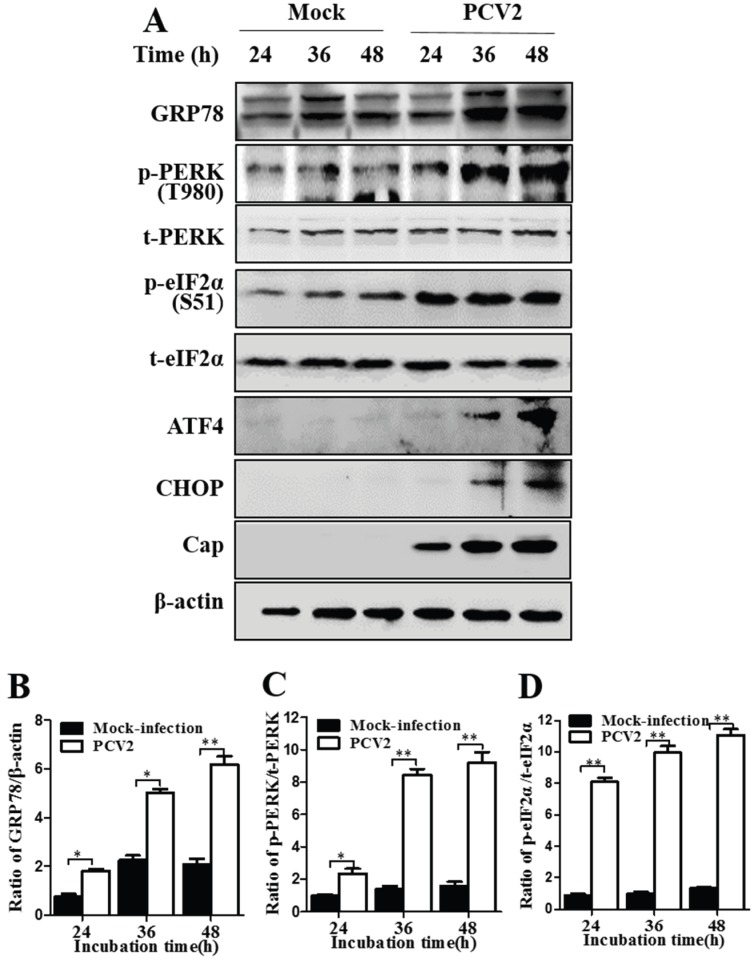
Porcine circovirus 2 infection induced unfolded protein response and expression of the pro-apoptotic protein CHOP by activating the PERK/eIF2α/ATF4/CHOP axis. PK-15 cells were infected with PCV2 or mock-infected for indicated time points. (**A**) Representative images of Western blotting for the target proteins; (**B**–**D**) Ratios of GRP78 to β-actin (**B**), phosphorylated PERK (p-PERK) to total PERK (**C**), and phosphorylated eIF2α (p-eIF2α) to total eIF2α (t-eIF2α) (**D**). Ratios of targeted proteins to β-actin or t-PERK/t-eIF2α were normalized to mock infection set at 1.0. All data are reported as means ± the standard errors of the mean of three independent experiments (*, *p* < 0.05; **, *p* < 0.01) here and in all subsequent figures.

Next, we made a semi-quantitative approach to investigate changes of p-PERK, p-eIF2α and the ER chaperone GRP78 at 24, 36 and 48 hpi. GRP78 was investigated because it is known not only as a marker of ER stress but also an important regulator of ER stress by modulating the activation of transmembrane ER stress sensors (IRE1, PERK, and ATF6) through a binding-release mechanism [17,21]. Figure 2 shows that p-PERK, p-eIF2α and GRP78 were induced progressively during PCV2 infection and remained elevated at 48 hpi as compared to mock-infected cells (*p* < 0.05 or <0.01 at all time points). The profiles of these molecules were similar to the kinetics of the viral capsid protein expression (Figure 2). The above results suggest that the viral protein synthesis during PCV2 infection induces UPR via activation of PERK/eIF2α.

ATF4 is a master regulator that plays pivotal roles in stress responses by regulating expression of many genes including *chop* that encodes the C/EBP-homologous protein (CHOP) by binding its promoter [32]. PERK is known to activate the eIF2α/ATF4 pathway [17]. Therefore, we attempted to analyze ATF4 and CHOP during PCV2 infection. Figure 2A shows that PCV2 infection also led to increased expression of ATF4 and CHOP at 36 and 48 hpi, but not at 24 hpi when PERK/eIF2α activation became significant. There was an apparent time gap between the initial UPR response and activation of its downstream molecules. Both ATF4 and CHOP were barely detectable in the lysates of mock-infected control cells. These results suggest a possible mechanistic link between induction of the pro-apoptotic CHOP and PCV2-induced apoptosis.

Because monocyte/macrophage lineage cells, including alveolar macrophages, are the major target cells for PCV2 [33], we attempted to examine if UPR was activated in the porcine alveolar macrophage cells infected with PCV2. We found similar results to those in PK-15 cells, no activation of IRE1 and ATF6, but activation of PERK-eIF2α as well as increased GRP78 expression and LC3 lipidation, as compared with the mock control (Figure S1).

### 3.2. Inhibition of PERK and eIF2α Dephosphorylation Reduced PCV2 Replication

**Figure 3:**
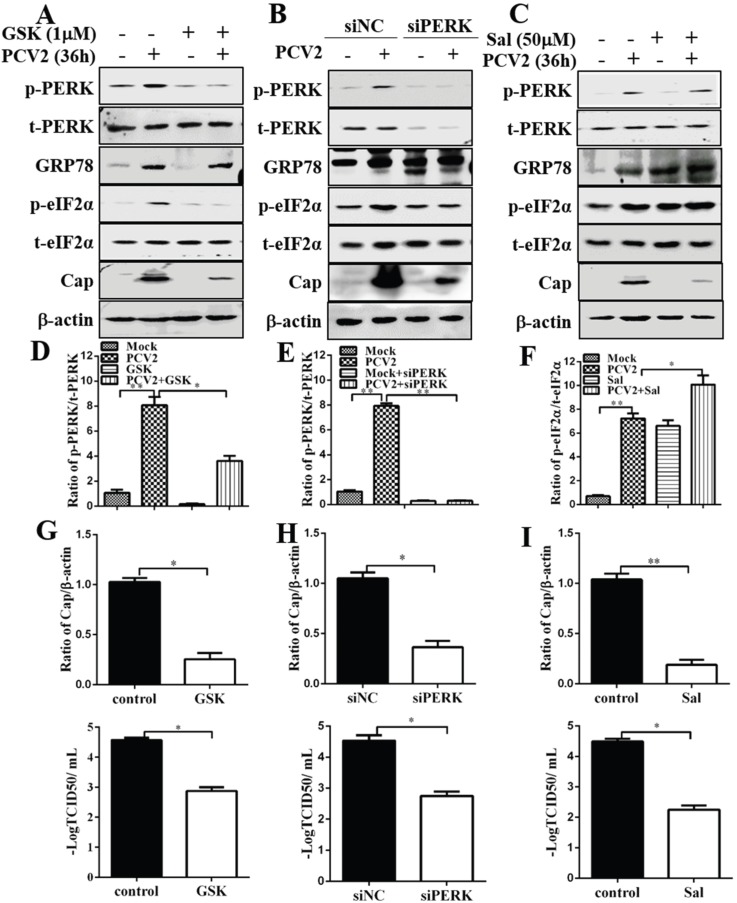
Inhibition of PERK by GSK2606414 or by RNA interference and attenuation of eIF2α dephosphorylation by salubrinal reduced porcine circovirus 2 replication. PK-15 cells were infected with PCV2 (MOI = 1) for 36 h in the presence of GSK2606414 (GSK, 1 μM) or salubrinal (Sal, 50 μM). PK-15 cells were first transfected with PERK-specific siRNA (siPERK). Scrambled RNA was used as control. After 24 h of transfection, the cells were infected with PCV2 or mock-infected for 36 h. Whole cell lysates were subjected to Western blotting. (**A**–**C**) Representative blots of PCV2-infected cells treated with GSK2606414, siPERK and salubrinal respectively; (**D**,**E**) Inhibition of PERK by GSK2606414 or siPERK (ratio of p-PERK to t-PERK); (**F**) Inhibition of eIF2α dephosphorylation by salubrinal (ratio of p-eIF2α to t-eIF2α); (**G**–**I**) Effects of p-PERK inhibition by GSK2606414 or RNA interference, and inhibition of eIF2α dephosphorylation by salubrinal on viral capsid (Cap) protein expression (ratio of Cap to β-actin normalized to control cells set at 1.0, top panels) and virus titer (log TCID_50_/mL, bottom panels).

Since PCV2 infection could activate the PERK pathway, we wanted to examine if inhibition of PERK with GSK2606414, a selective PERK inhibitor [34] or by RNA interference would have effects on the expression of GRP78, eIF2α or even viral capsid protein. Figure 3 shows that GSK2606414 treatment and siPERK not only reduced p-PERK induced by PCV2 infection but also decreased p-eIF2α (Figure 3A,B,D,E). PERK inhibition did not seem to have effect on GRP78 expression. However, viral replication was significantly reduced, as revealed by reduced expression of Cap and lower virus titers (Figure 3A,B,G,H). To determine the effect of eIF2α phosphorylation level on viral replication, we treated PCV2-infected cells with salubrinal, a selective small molecule inhibitor of cellular complexes (containing the protein phosphatase 1 and its cofactor GADD34) that dephosphorylates eIF2α and inhibits global translation [35]. We found that p-eIF2α was significantly increased by salubrinal treatment in PCV2-infected cells compared with control cells, while the total eIF2α remained unchanged (Figure 3C,F). GRP78 level was also elevated (Figure 3C). Inhibition of elF2α dephosphorylation significantly reduced Cap expression and virus titers (Figure 3C,I). These results indicate that PCV2 may utilize the PERK pathway for its replication.

### 3.3. GRP78 Positively Regulated PCV2 Replication

**Figure 4:**
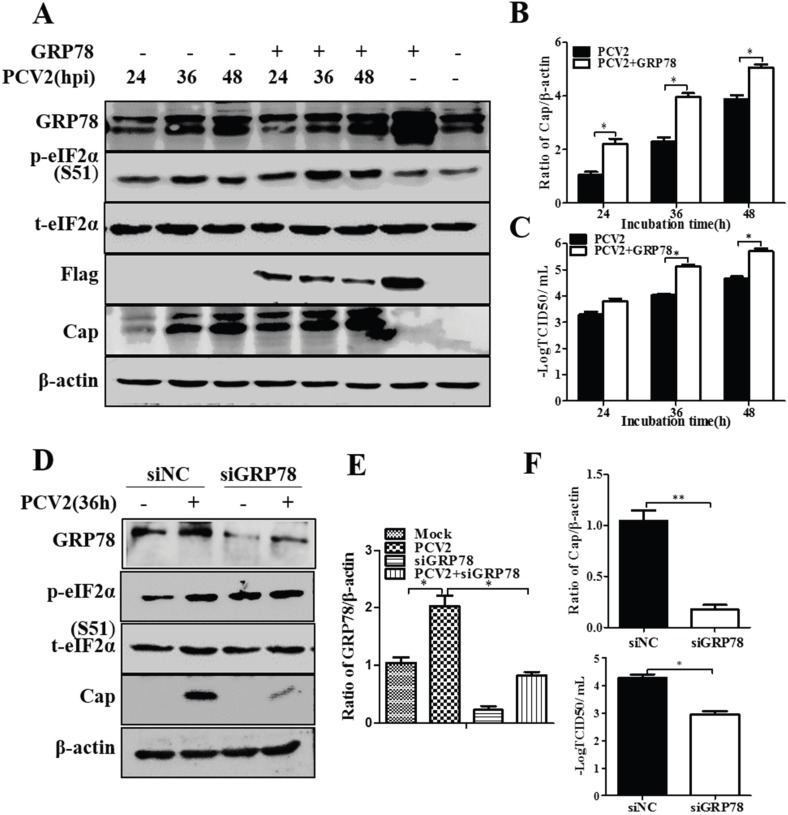
Effects of GRP78 over-expression or knockdown on porcine circovirus 2 replication. PK-15 cells were first transfected with the vector expressing GRP78 or with GRP78-specific siRNA (siGRP78). Control vector pcDNA or scrambled RNA (siNC) was used as control. After 24 h of transfection, the cells were infected with PCV2 or mock-infected for indicated time points. Whole cell lysates were subjected to Western blotting for GRP78, phosphorylated eIF2α (p-eIF2α), total eIF2α (t-eIF2α), Flag tag, and viral capsid protein (Cap). (**A**) Representative blot showing the effects of GRP78 over-expression on target proteins; (**B**) Ratio of Cap to β-actin normalized to mock infection set at 1.0; (**C**) Virus titers expressed as log TCID_50_/mL; (**D**) Representative blot showing the effects of GRP78 silencing on target proteins; (**E**) Ratio of GRP78 to β-actin; (**F**) Ratio of Cap to β-actin normalized to control cells set at 1.0 (top panel) and virus titers expressed as log TCID_50_/mL (bottom panel).

One of the key events in UPR activation is dissociation of the chaperone GRP78 from the luminal part of the ER integral membrane proteins: PERK, IRE1 and ATF6 [17]. Over-expression of GRP78 has been shown to increase ER resistance to stress, thus having beneficial effects in several cell types [36,37]. We transfected the PK-15 cells with a recombinant eukaryotic vector pcDNA3-GRP78 which was Flag-tagged for distinction between endogenous and exogenous forms of GRP78. Extrachromosomal transient expression was successful as shown by the presence of Flag on the blots with the GRP78 level particularly high in the cells not infected with PCV2 (Figure 4A). Overexpression of GRP78 did not seem to have observable effect on p-eIF2α (Figure 4A). Viral protein synthesis (Figure 4A,B) and virus titers increased significantly (*p* < 0.05) (Figure 4C) as a result of pcDNA3-GRP78 transfection. It has been shown that knockdown of GRP78 could activate UPR [38]. We silenced GRP78 expression using RNA interference. The effect of GRP78 silencing was confirmed by Western blot (Figure 4D,E). Silencing of GRP78 led to increased phosphorylation of eIF2α in mock-infected cells, but did not affect p-eIF2α in PCV2-infected cells (Figure 4D). However, such knockdown resulted in decreased viral protein synthesis and the virus titers (Figure 4D,F).

**Figure 5:**
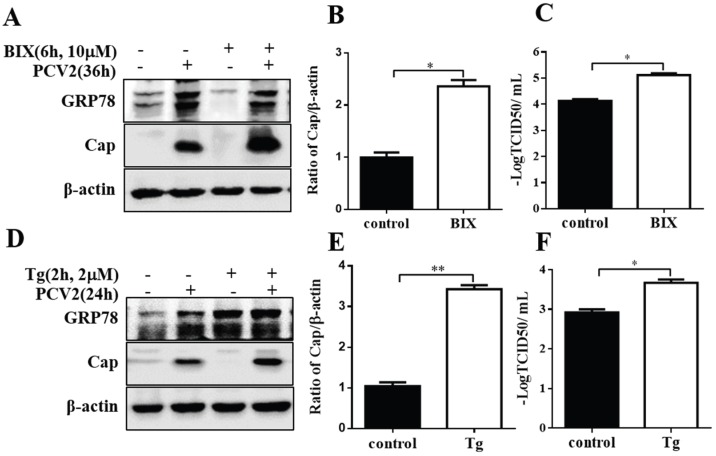
Induction of GRP78 by pretreatment with BIX or thapsigargin enhanced viral capsid protein expression. PK-15 cells were pre-treated with 10 μM GRP78 inducer BIX for 6 h or with 2 μM thapsigargin (Tg) for 2 h and then infected with PCV2 (MOI = 1) in fresh medium for another 36 h or 24 h. Whole cell lysates were then subjected to Western blotting for GRP78, Cap and β-actin. (**A**,**D**) Representative blot showing the effects of GRP78 induction by BIX or thapsigargin on viral capsid (Cap) protein expression; (**B**,**E**) Ratio of Cap to β-actin normalized to control cells set at 1.0; (**C**,**F**) Effects of GRP78 induction by BIX or thapsigargin on virus titer expressed as log TCID_50_/mL.

To further elucidate the effect of GRP78 on PCV2 replication, we induced GRP78 by pre-treatment of the cells with chemical compounds BIX [39] and thapsigargin (Tg). Treatment of cells with BIX for 6 h significantly increased GRP78 expression. Six-hour pre-treatment with BIX significantly induced viral protein synthesis and virus titers in PCV2-infected cells (*p* < 0.05) (Figure 5A–C). The GRP78 level of the pretreated group fell to the normal level during subsequent infection for 36 h, which is not surprising since the effect of BIX is known to be transient [39]. We showed that thapsigargin was able to rapidly induce UPR in PK-15 cells (Figure S2). Pre-incubation with this compound for 2 h also increased viral protein synthesis and virus titers (*p* < 0.05) (Figure 5D–F). The same treatment performed on porcine alveolar macrophage cells gave similar results.

### 3.4. Chemical ER Chaperones Modulated PCV2 Replication and Apoptosis

**Figure 6:**
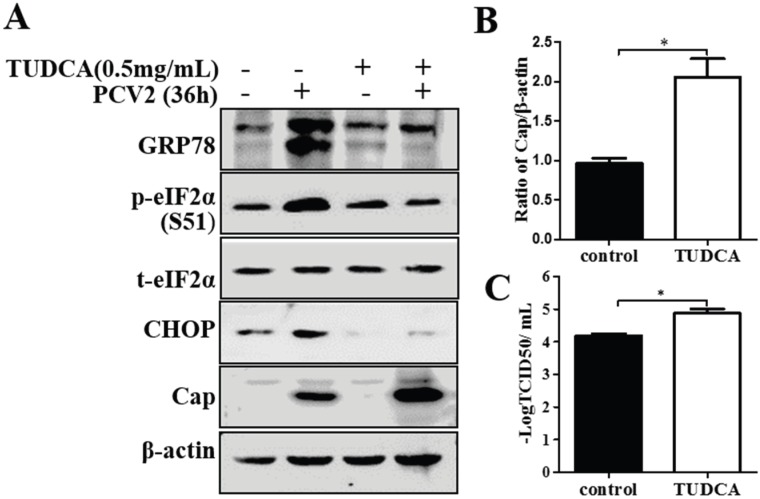
Tauroursodeoxycholic acid enhanced virus replication. PK-15 cells were infected with PCV2 (MOI = 1) for 36 h. Tauroursodeoxycholic acid (TUDCA) was maintained during the viral infection. Whole cell extracts were subjected to Western blotting for GRP78, phosphorylated eIF2α (p-eIF2α), total eIF2α (t-eIF2α), CHOP and capsid protein. (**A**) Representative blot showing the effect of TUDCA treatment on expression of target proteins; (**B**) Ratio of Cap to β-actin normalized to untreated control cells set at 1.0; (**C**) Virus titers expressed as log TCID_50_/mL.

To further understand the role of UPR in the pathogenesis of PCV2 infection, we examined the effects, on viral replication and virus-induced apoptosis, of chemical chaperones that modulate ER responses to stress. TUDCA is one of the molecules that have been shown to reduce ER stress *in vitro* and *in vivo* [40]. We found that treatment with TUDCA reduced GRP78 expression, eIF2α phosphorylation and CHOP expression induced by PCV2 infection (Figure 6A). TUDCA increased viral capsid protein expression and virus yield (Figure 6A–C). The TUNEL assay showed that PCV2 had actually induced apoptosis by 36 hpi, while TUDCA reduced PCV2-induced apoptosis. Another chemical chaperone 4-phenylbutyric acid (a low molecular weight fatty acid), which is known to alleviate ER stress, decreased p-eIF2α and CHOP in PCV2-infected cells. However, it was inhibitory to viral protein expression and virus yield (Figure S3). Treatment of PCV2-infected PK-15 with 4-PBA also reduced apoptotic cells, coincident with down-regulation of CHOP.

### 3.5. Pharmacological Treatments, Plasmid Transfection and RNA Interference Did Not affect Cell Viability

**Figure 7:**
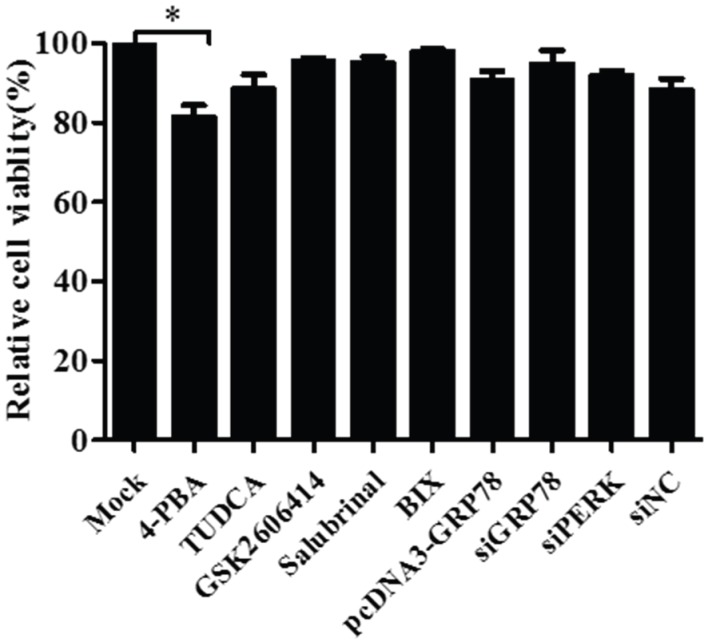
Pharmacological treatments or plasmid transfection and siRNA knockdown did not affect cell viability with the exception of 4-phenylbutyric acid. Cell viability was determined by CCK-8 assay after treatment of the PK-15 cells with 0.5 mg/mL TUDCA, 10 mM 4-PBA, 1 μM GSK2606414, 50 μM salubrinal, 10 μM BIX or transfection with pcDNA3-GRP78, siGRP78, siPERK or scrambled RNA (siNC) for 24 h. Relative percent cell viability is expressed as means ± SEM (*n* = 3).

To assess whether treatments with TUDCA, 4-PBA, GSK2606414, salubrinal or BIX and transfection with plasmids for GRP78/PERK-siRNA or GRP78 over-expression affect cell viability, which could ultimately impact experimental results in PK-15 cells, we used the CCK-8 assay to analyze their effects on cell viability. Transfection or treatment with chemicals did not have significant effects on the viability of PK-15 cells with the exception of 4-PBA (Figure 7).

## 4. Discussion

A host of RNA viruses have been found to trigger ER stress and differentially activate ER stress-related signaling pathways [41]. However, there is paucity of information on the involvement of DNA viruses in ER stress. Herpes simplex virus-1 was found to disarm UPR in early stages of infection, but induced eIF2α/ATF4 signaling at the final stage of its replication [42]. With Varicella-zoster virus, a DNA virus that possesses the smallest genome of human herpesviruses and lacks some genes used by other herpesviruses to manipulate the UPR, differential induction of UPR was seen as its strategy to cope with viral glycoprotein synthesis [23]. However, it remains unknown if single stranded DNA viruses, such as circovirus, are able to manipulate UPR during their infection. Here we show that PCV2 could employ UPR to enhance its replication via PERK/eIF2α signaling.

Viruses differ in their ability to activate the three arms of UPR transmembrane sensors, PERK, IRE1 and ATF6, some activating all three while some one or two during their infection [41]. Theoretically, up-regulation of GRP78 in response to stresses or viral infection could be perceived by all three UPR sensors with subsequent activation [43]. First, we demonstrated that PCV2 infection did initiate UPR by inducing expression of GRP78, the master regulator of the UPR pathways, both in PK-15 and porcine alveolar macrophage cells. An early study has indicated that alveolar macrophages are the major target cells for PCV2 [33]. We also found that the PAM cells are permissive to PCV2 replication. Therefore, the findings in this particular cell line, though similar to those in PK-15 cells showing induction of UPR and autophagic responses by PCV2 infection, should strengthen the view that both UPR and autophagy could be involved in the pathogenesis of PCV2.

Then, we examined all three arms of UPR during PCV2 infection. It is evident that the PERK pathway was selectively activated without concomitant activation of the IRE1/XBP1 or ATF6 pathway. In the PCV2-infected cells, there were actually two bands of GRP78, the lower one at about 60 kDa being more significantly induced. Because the anti-GRP78 monoclonal antibody recognizes the C-terminus of GRP78, we speculate that this lower band could be an isoform of GRP78 lacking ER-targeting signal in the N-terminus, as reported by Ni *et al.* [44]. They found that the cytosolic isoform of GRP78 with a molecular weight of about 62 kDa was expressed in several cell types as a result of alternative splicing and involved in PERK activation by antagonizing the PERK inhibitor P58IPK, a DnaJ family protein that could inhibit the PERK kinase activity. This might explain why PCV2 infection activates PERK/eIF2α only.

The PERK/eIF2α pathway is required not only for global translational attenuation upon ER stress, but also for transcriptional activation of genes that critically regulate UPR or apoptosis [45]. The pro-survival transcriptional factor ATF4 could contribute to the expression of genes involved in remediation of cellular stress damage, including expression of pro-apoptotic CHOP [32]. We found that the pro-survival transcriptional factor ATF4 in PCV2-infected cells was induced probably via selective up-regulation by PERK/p-eIF2α [46]. Both ATF4 and CHOP were induced at a later time point than PERK/eIF2α (36–48 hpi *vs.* 24 hpi) when the viral capsid protein expression was significantly elevated and remained high. Therefore, we propose that persistent PCV2 infection could lead to selective activation of PERK and its downstream molecules via the PERK-eIF2α-ATF4-CHOP axis [47].

Attenuation of eIF2α dephosphorylation by salubrinal inhibited PCV2 replication. This may be attributed to suppression of global protein translation, including viral protein synthesis, due to elevation of p-eIF2α [41]. It has been reported that salubrinal has antiviral activity against herpes simplex virus-1, hepatitis C virus and dengue virus [35,41,48]. However, when PERK phosphorylation was inhibited by GSK2606414, viral replication was suppressed irrespective of reduction of p-elF2α. It remains unknown if GSK260414 itself is antiviral. These results indicate that PCV2 may utilize the PERK pathway for its replication.

GRP78 plays a dual role in the ER by mediating protein folding to prevent aggregation and by activating the UPR signaling [43]. Over-expression of GRP78 increased PCV2 replication while GRP78 silencing had an opposite effect on the virus. Both treatments did not seem to have apparent effects on p-eIF2α, suggesting that GRP78 might be involved in assisting folding of viral proteins for PCV2 replication. We then induced GRP78 by 6-h treatment with BIX or 2-h treatment with thapsigargin prior to PCV2 infection. Both treatments enhanced viral protein expression possibly as a result of GRP78 induction. Although we did not find marked increase of GRP78 expression in cells pretreated with BIX followed by PCV2 infection for 36 h, we did see dose-dependent increase of GRP78 expression during 6-hour BIX treatment. It has been shown that the effect of BIX on GRP78 induction is transient [39]. Nevertheless, it is possible that GRP78 might have contributed to PCV2 replication by its cytoprotective effect, as have been observed in Ebola and betanodavirus viruses [49,50]. These findings suggest that GRP78 is utilized by PCV2 for its replication in PK-15 cells.

Chemical chaperones, such as TUDCA and 4-PBA, are compounds known to alleviate UPR by assisting in protein folding and preventing protein aggregation [51,52]. In our study, both chemicals were found to down-regulate the expression of GRP78, p-eIF2α and CHOP (although TUDCA was more effective), suggesting their roles in lessening UPR in PCV2-infected cells. With the TUNEL assay, we observed that treatment of PCV2-infected cells with TUDCA or 4-PBA also decreased apoptosis probably as a result of down-regulation of the pro-apoptotic protein CHOP (data not shown). In this sense, we tend to believe that there is link between PCV2-induced UPR and apoptosis. Concerning their effects on PCV2 replication, an opposing scenario was seen: TUDCA enhanced viral replication while 4-PBA was inhibitory to the virus. Cell cycle arrest by 4-PBA treatment [53] could explain its inhibitory effect on PCV2 because its replication was dependent on S- and G2/M phase [54]. Several reports indicate that TUDCA repressed replication of a number of viruses, such as respiratory syncytial virus, influenza A virus, hepatitis C virus and tick-borne encephalitis virus [55,56,57]. However, we show for the first time that TUDCA actually supported PCV2 replication. There are two possibilities. One could be that TUDCA assists in proper folding of the PCV2 capsid protein and prevents its aggregation in the cytoplasm [51] such that the protein could be shuttled effectively into the nuclei for virus assembly [58]. The other might be that the anti-apoptotic effect of TUDCA provides a favorable cell condition for viral replication.

**Figure 8:**
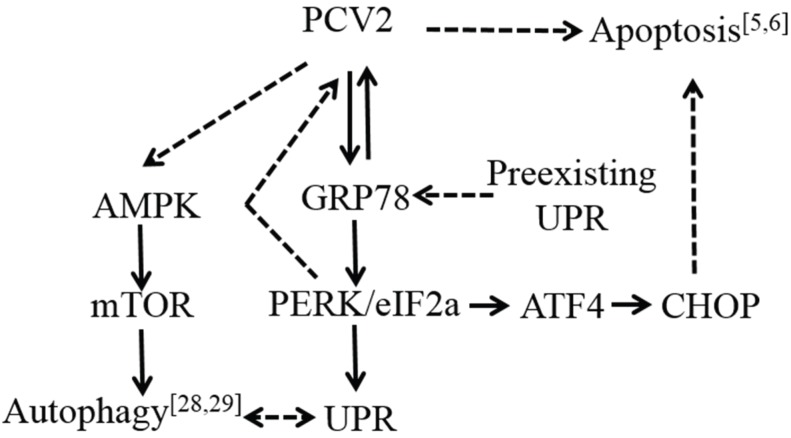
A schematic model of cellular responses during porcine circovirus type 2 infection. Arrows with solid lines indicate induction, while those with dashed lines suggest that the molecules or pathways remain to be investigated.

In summary, we conclude that PCV2 infection is capable of triggering UPR via selective activation of the PERK pathway and that endogenous chaperone GRP78 and chemical chaperone TUDCA could enhance PCV2 replication. Our findings in this report and earlier publications provide clear evidence that PCV2, though small and simple, could induce multifaceted host cell responses of UPR, autophagy and apoptosis (Figure 8). Warranted for further research is to decipher if these cellular events are intertwined, what is the principal triggering factor(s) and how these cellular events are regulated during PCV2 infection. Such work may lead to better understanding of PCV2 pathogenesis.

## 5. Conclusions

PCV2 could induce ER stress in its infected cells by selective activation of PERK and its downstream molecules via the PERK-eIF2α-ATF4-CHOP axis and might deploy UPR and ER chaperone GRP78 to enhance its replication. Therefore, we propose that ER stress plays a role in the pathogenesis of PCV2 infection probably as part of autophagic and apoptotic responses.
